# Measurement invariance of the Hopkins Symptoms Checklist: a novel multigroup alignment analytic approach to a large epidemiological sample across eight conflict-affected districts from a nation-wide survey in Sri Lanka

**DOI:** 10.1186/s13031-017-0109-x

**Published:** 2017-04-26

**Authors:** Alvin Kuowei Tay, Rohan Jayasuriya, Dinuk Jayasuriya, Derrick Silove

**Affiliations:** 1 The Academic Mental Health Unit, Psychiatry Research and Teaching Unit, Liverpool Hospital; School of Psychiatry, University of New South Wales, Cnr Forbes and Campbell Streets, Liverpool, NSW 2170 Australia; 2 Community Medicine and Public Health, Faculty of Medicine, University of New South Wales, Sydney, NSW Australia; 3 Development Policy Centre, Australian National University, Canberra, Australian Capital Territory (ACT) Australia

## Abstract

**Background:**

The alignment method, a novel psychometric approach, represents a more flexible procedure for establishing measurement invariance in geographically, ethnically, or linguistically diverse samples, especially in large epidemiological surveys. Although the Hopkins Symptoms Checklist (HSCL-25) has been used extensively in the field to assess anxiety and depressive symptoms, questions remain about the comparability of findings when the instrument is applied across regions in large-scale national surveys.

**Methods:**

The present study is the first in the field to apply the alignment method to test the structure and measurement invariance of the anxiety and depression dimensions of the HSCL-25 amongst Sri Lankan subpopulations (*n* = 8456) stratified by geographical regions, levels of past exposure to conflict, and ethnic composition.

**Results:**

Multigroup CFA analysis yielded non-converging models requiring substantial modifications to the models. As a result, multigroup alignment analysis was applied and the results supported the bifactorial structure and measurement invariance of the HSCL-25 across eight (severe and moderate) conflict-affected districts. The alignment analysis based on a good-fitting configural model yielded a metric non-invariance of 22.22% and scalar non-invariance of 5.88% (both under the established 25% threshold). The bifactorial model outperformed the tripartite and other models. In comparison to the anxiety items, the depressive items showed higher levels of metric non-invariance across districts.

**Conclusions:**

Our findings demonstrate the methodological feasibility of applying the alignment method to test the structure and invariance of the HSCL across ethnically diverse populations living in conflict-affected districts in Sri Lanka. Further studies are needed to examine ethnicity and language factors more critically.

**Electronic supplementary material:**

The online version of this article (doi:10.1186/s13031-017-0109-x) contains supplementary material, which is available to authorized users.

## Background

Epidemiological studies undertaken across diverse settings in the post-conflict field have shown high prevalence rates of anxiety and depressive symptoms, the most commonly assessed mental health outcomes together with posttraumatic stress symptoms [1]. Although the Hopkins Symptoms Checklist (HSCL-25) has been used extensively in the field to assess anxiety and depressive symptoms [2], questions remain about the comparability of findings when the instrument is applied across regions, for example, in large-scale national surveys. Within the context of the field of transcultural mental health traumatology, measurement invariance allows assessment of the extent to which the construct under study is being understood and interpreted in a similar manner by respondent populations that may differ in cultural, ethnic, and linguistic backgrounds [3]. Establishing measurement invariance will indicate whether it is legitimate to compare responses on the anxiety and depression subscales of the HSCL-25 across different populations within the broader society.

The conventional first step in testing measurement invariance is to assess the configural component, that is, whether the relationship between observed indicators (symptoms) and underlying latent factors is uniform across different subpopulations [4]. Other indices that can be tested subsequently include metric invariance (equivalence in factor loadings) and scalar invariance (equivalence in intercepts), although the debate continues as to whether these tests represent too strict a standard to judge invariance [5]. In that regard, it is increasingly acknowledged that the commonly applied method, multigroup confirmatory factor analysis (MGCFA) in which scalar invariance is required to compare latent mean scores across groups, may set too stringent a standard for testing invariance [6]; in particular, the method may not be suitable for testing invariance across a large number of subgroups given the complexity and the extent of the modifications commonly required to achieve invariance [7]. In that regard, MGCFA involves iterative testing of an increasingly restrictive set of factorial models, commencing with the configural model, and then progressing to models that hold relevant parameters to be equal (factor loadings, intercepts, factor variances, residual variances) [4]. Invariance achieved in factor loadings (referred to as metric invariance) and intercepts (referred to as scalar invariance) suggests that item responses are interpreted and understood in a uniform manner across groups, a prerequisite for comparing group differences [5]. Put simply, measurement invariance implies that the construct being measured by an instrument is understood and responded to in an equivalent manner across two or more groups. If measurement variance is found, this means that there are fundamental quantitative and/or qualitative differences in the construct or the procedure being used to measure it across study groups, disparities that may be attributed to metric differences (differential item loadings on factorial solutions) or scalar variance (differential intercepts or response styles). However, it has been argued that the requirements of metric and scalar invariance as specified within MGCFA may be overly restrictive particularly when comparing inter-individual or between-group differences in mental health reports across cross-cultural groups, given that it is expected that responses will vary to some extent according to individual and cultural influences [7, 8].

The alignment method, a novel approach developed and tested in a large cross-country survey [8], represents a more flexible procedure for establishing invariance when a number of subpopulations (for example, residing in different regions) are included in the composite sample. In contrast to MGCFA, the alignment modelling approach allows for an examination of inter-individual and between-group differences that influence variance (which may be related to the comprehension and interpretation of the measure) across a large number of groups that differ in demographic and other characteristics [8].

The HSCL-25 has been used extensively across clinic and community settings amongst culturally diverse samples of asylum seekers [9], refugees [10], and other post-conflict populations in high and low-medium income countries (LMICs) [2]. The measure has been adapted and translated for use in conflict settings in Asia [11–13], the Middle East [14], Africa [15, 16], and the former Yugoslavia [17, 18]. The HSCL-25 is currently available in a wide range of languages including Arabic [19], Hmong [20], Kiswahili [16], Pashto [14], Farsi, Dari, Bosnian, Somali [9], Vietnamese [21], Swedish [22], Serbo-Croatian, Russian [23], Tibetan [13], Indochinese [24, 25], and Khmer.

A substantial body of research, including convergence studies comparing the HSCL with structured clinical interviews, has provided broad support for the cross-cultural validity and psychometric properties of the HSCL [11, 13, 14, 17, 25]. For example, there is evidence of sound internal consistency for the entire scale (Cronbach’s α generally exceeding 0.90) and for the subscales of depression (0 .85) and anxiety (0.76) [9, 16, 19, 23, 26]. The bi-factorial structure (anxiety and depressive symptoms) has been supported by studies across diverse cultures, for example, for Southeast Asia [20] and Afghanistan [14]. A recent item response analysis conducted by Haroz and colleague [27] based on the HSCL-15 supported the cross-cultural equivalence of depression symptoms amongst ethnically and linguistically diverse conflict-affected populations from eight low-income countries (Colombia, Indonesia, Iraq, Rwanda, Kurdistan Iraq, Thailand, and Uganda). In addition, although all items showed some degree of differential item functioning (DIF), Indonesia being the only country where the prevalence estimate of depression could have been overestimated due to possible measurement variance [27].

At the same time, other studies focusing on the HSCL in high-income, Anglophone countries have found support for a tripartite factorial model [28, 29] including (in addition to anxiety and depression) a mixed domain of symptoms, variously labelled as “general/mixed distress”, “autonomic anxiety”, and “somatic depression.” Nevertheless, greatest consistency has been found in the association between potentially traumatic events (PTEs) and ongoing adversities typical of post-conflict populations, with the HSCL-25 anxiety and depression scales, respectively, with some minor differences in these relationships between the two symptom domains [9, 15, 30, 31].

Extant studies investigating the measurement invariance of the HSCL-25 have been restricted to small and non-representative samples [23, 32], often comparing different countries [9] where the constituent populations have been exposed to widely differing conditions and traumatic events. Remarkably, no studies have investigated the measurement invariance of the anxiety and depressive domains of the HSCL-25 in a large, representative population sample in a post-conflict country.

The population of Sri Lanka has experienced a decades-long civil war waged between the government (GoSL) and the Liberation Tigers of Tamil Eelam (LTTE), a conflict that came to an end in 2009. During the prolonged period of violence, there was extensive physical injuries and deaths, mass displacement of whole populations, and extensive deprivations, including of food, water, and medical care [33]. Prior to the conclusion of the armed conflict, the LTTE claimed a large portion of the territories in the north-eastern region of Sri Lanka, forming a de facto state, with its administrative capital situated in Kilinochchi. By the end of the conflict in 2009, over 36% of the entire population of the north was displaced, including virtually all civilians of the former LTTE controlled areas (Mullativu, Killinochi) [34]. Within three years, 236,429 (90%) of the internally displaced persons (IDPs) had returned to their homes [35]. Sinhalese and Sri Lankan Tamils represent the two largest ethnic groups in Sri Lanka, numbering approximately 74 and 12 percent of the population respectively, with other minority groups comprising Indian Tamils (6%) and Sri Lankan Muslims/Moors (9%) [36].

The historical and demographic context of Sri Lanka offered an opportunity to test the measurement invariance of the anxiety and depressive symptom dimensions of the HSCL-25 amongst subpopulations that differed in ethnic composition, first languages, and regional exposure to conflict. Given these distinctive aspects across different subpopulations, it is imperative to assess for possible measurement variance when comparing the prevalence of depressive and anxiety symptoms at a district or regional level. Our objective was to apply a novel statistical approach, the multigroup alignment method, to test the bifactorial structure and measurement invariance of the anxiety and depression dimensions of the HSCL-25 amongst Sri Lankan subpopulations stratified by geographical regions, levels of past exposure to conflict, and ethnic composition.

## Methods

### Sample

Our study draws on mental health data collected during a representative survey (*n =* 20,632) conducted during February through April, 2014 across Sri Lanka. The primary purpose of the study was to gather data about migration intentions, the mental health component being added as a discrete component. Details of the study have been published elsewhere [37]. In summary, a multi-stage sampling design was used, covering all districts of Sri Lanka that were exposed to conflict (*n =* 8), nine districts randomly selected from the remaining 16, and Colombo, the capital.

Sampling units were selected at the second lowest administrative level (Grama Sevaka, DS or Divisional Secretary’s Division) using the probability proportion to size (PPS) method based on national census data gathered in 2012. Eight DSs were selected for large districts and four for small districts (smaller districts were defined as those with fewer than 4 DSs). Next, we selected units of the lowest administrative level (Grama Niladari, GN, also known as “village officer”), using PPS. Five GNs were selected within each DS for large districts, and 10 GNs were selected within each DS for small districts. Finally, we randomly selected 28 households at the GN level, and randomly selected an adult household member within the dwelling. The response rate from the 26,600 people approached was 81%.

## Measures

### Hopkins Symptoms Checklist (HSCL)

We applied the Hopkins Symptoms Checklist (HSCL-25) [24], a 25-item cross-culturally validated measure of depression and anxiety symptoms used extensively amongst post-conflict and refugee populations worldwide [23]. The HSCL-25 has been translated into Tamil for a study in the north of Sri Lanka [38] and amongst asylum seekers in Australia [39, 40]. We translated the measure to Sinhalese, an Indic language spoken by the Sinhalese who form the majority of the Sri Lankan population. Translations followed accepted international procedures for translation and back translation [41]. Psychometric testing of the HSCL across culturally distinct populations from Sub-Saharan Africa [16], Eastern Europe [17], and Asia [11, 13, 25, 42] yielded sound internal consistency (Cronback’s alpha ≥ .90 for the entire scale; ≥.85 for the depression subscale, ≥.76 for the anxiety subscale), inter-rater reliability (intra-class r ≥ .80), and test-retest reliability (≥.80) for the scale as a whole. Respondents rated each symptom according to the conventional four-point frequency scale (1 = not at all, 2 = a little, 3 = quite a lot, 4 = extremely). In the present study, the HSCL-25 was tested for its ease of administration in a pilot study of 1000 persons including all relevant ethnic groups. In addition, we found a sound level of test-retest reliability for the HSCL-25 amongst a random subsample (*n =* 1000) of respondents from the present sample re-interviewed following the full survey (depression subscale: Kappa = 0.80; anxiety subscale: Kappa = 0.85; full measure: 0.89). We defined “symptomatic depression” and “symptomatic anxiety” according to the conventional international cut-off scores of >1.75 for each subscale.

### Personnel and training

Members of the research team trained local field workers (*n =* 83) in applying the measures using an electronic platform. The interviews were conducted in the home language (either Sinhala or Tamil) in strict privacy and responses were entered directly into tablet devices. Data were accessed daily by the lead survey manager alone.

### Statistical analysis

We stratified districts by severity of conflict based on the extent of exposure to the most recent episode of war (2008–2009) and the level of population displacement (>75%), information accessible from national statistical data [34]. We thereby derived two broad groupings, severe conflict/displacement areas (Mannar, Kilinochchi, Mullaitivu) and moderate conflict areas (Jaffna, Batticaloa, Trincomalee, Vavuniya, Puttalam), collectively including 8456 persons. In addition, we further subdivided the sample by ethnicity (Sinhalese, Tamils, and Moors/Burghers).

We calculated descriptive statistics in relation to sociodemographic variables and prevalence of anxiety and depressive symptoms, stratified by districts of high conflict exposure ((Mannar, Kilinochchi, Mullativu) and moderate conflict exposure (Jaffna, Batticaloa, Trincomalee, Vavuniya, Puttalam). Puttalam (23.5%) had the lowest population displacement ratio compared to the other moderate conflict areas and was used as the reference group. We made comparisons using chi-square statistics adjusted for sampling weights (F-adjusted tests).

The first step of the alignment analysis involves testing a configural model (base model) in which all intercepts and loadings are unconstrained, with the factor means and variances fixed to 0 and 1 respectively [8]. The second step involves optimization of the measurement parameters (factor loadings, intercepts/thresholds) allowing an optimal invariance pattern to be identified based on minimum non-invariant parameters using a simplicity function similar to the rotation criteria of exploratory factor analysis (EFA). The simplicity function (F) represents the amount of accumulated measurement non-invariance whose contributions can be isolated for each variable (i.e. smaller simplicity function contributes to greater level of invariance) with the ultimate goal of locating the optimal solution that minimizes the simplicity function. The third step involves adjustment of the factor means and variances according to the optimal alignment, analogous to the rotated model of EFA [7]. In addition, we compared the fit of a series of alignment models tested using the fixed alignment approach in with the FIXED alignment approach in which the factor mean was fixed to 0 in the reference group (represented by Puttalam). We tested the same models using the FREE method (all factor means were freely estimated) which was poorly identified and therefore FIXED method (with Putalam fixed as the reference category) was used to estimate the model. The FIXED alignment optimization method is recommended in instances of minimal metric non-invariance, a condition commonly occurring in an analysis of a small number of groups [7].

Given that our focus was on the HSCL-25 scales of anxiety and depression, the most widely used indices in the field, the base configural model tested specified that these two dimensions loaded on their respective latent factors. In addition, however, we tested a three-factor model based on the tripartite model proposed by Clark and Watson (1991) defined by the core constellations of anxiety and depressive symptoms with an additional cluster for non-specific symptoms of insomnia, fatigue, restlessness, weakness, and feeling tense [28]. Prior to the alignment analysis, our Multigroup CFA analysis based on the bi-factorial and three-factorial models failed to support metric invariance across groups. Given the large number of modifications required to potentially achieve convergence, we did not pursue this approach further.

In order to examine for the effect of ethnicity, we tested the bifactorial and tripartite models on subsamples stratified by two ethnic groupings (Singhalese or the composite minorities, that is Tamils/Burghers/Moors). Each model was tested using the FIXED alignment optimization settings, a recommended approach that estimates all factor means. We used the lowest conflict district (Puttalam) as the reference category when testing the models using the FIXED setting.

We examined the Akaike and Bayesian information criteria to judge model fit, lower values indicating a better fitting model [5]. We calculated the degree of non-invariance based on the total number of measurement parameters (metric, scalar) multiplied by the number of groups and divided by the number of non-invariant parameters [8]. In addition, we examined adjusted residuals [(observed – expected) / √[expected x (1 + row total proportion) x (1- column total proportion)] of each item as a supplementary indicator of model misspecification.

Monte Carlo simulations performed previously on a large cross-country survey dataset indicated that an upper limit of 25% of non-invariant items provides evidence in support of measurement invariance of the measure as a whole [7]. Group-specific factor means were compared and rank-ordered for anxiety and depressive dimensions in the final stage following the step-wise alignment optimization procedure.

Given that we applied ordinal variables in our analyses, all models were estimated using WLSMV with numerical integration. The alignment analysis was adjusted for sampling weights, stratification, and clustering. Specifically, sampling weights were generated based on varying response rates at village level, over/under sampling across households, sex and ethnic representations (weighted according to the national census) across districts.

The analysis was performed in STATA version 14 [43] and Mplus version 7.2 [44].

## Results

### Sociodemographic characteristics across conflict-affected districts

Table 1 reports descriptive statistics for sociodemographic and mental health indices stratified by districts. Weighted chi-square tests indicated that the districts differed significantly in sociodemographic characteristics, ethnicity, exposure to displacement, and mental health indices. Notably, the severe conflict districts (Mannar, Killinochi, Mullativu) were more heavily populated by ethnic minorities including Tamils, Moors, and Burghers. Those residing in Mullativu and Killinochi also reported greater levels of displacement compared to the other districts. Depression based on the entire scale score was higher in Mullativu (17.2%) and anxiety in Killinochi (14.4%) and Batticola (14.4%), compared to the remaining populations.

**Table 1:** Descriptive analysis of sociodemographic variables stratified by 8 conflict-affected districts (*n =* 8456)

Characteristics	Jaffna *n =* 1051 (%)^a^	Mannar *n =* 1026 (%)	Vavuniya *n =* 1013 (%)	Mullativu *n =* 1076 (%)	Killinochi *n =* 1055 (%)	Battcaloa *n =* 1137 (%)	Puttalam *n =* 1112 (%)	Trincomalee *n =* 1016 (%)	*X* ^2^, P
Age group, years									
≥ 60	198 (18)	109 (9.9)	134 (12.2)	145 (13.2)	169 (15.4)	86 (7.8)	162 (14.7)	98 (8.9)	<0.000
51-60	163 (12.5)	170 (13)	173 (13.2)	163 (12.5)	173 (13.2)	172 (13.2)	161 (12.3)	133 (10.2)	<0.000
41-50	206 (12)	243 (14.1)	197 (11.4)	200 (11.6)	194 (11.3)	253 (14.7)	216 (12.5)	214 (12.4)	<0.000
31-40	270 (11.8)	272 (11.9)	269 (11.8)	295 (12.9)	272 (11.9)	305 (13.3)	308 (13.5)	297 (13)	<0.000
18-30	214 (10.4)	232 (11.2)	240 (11.6)	273 (13.2)	247 (12)	321 (15.5)	265 (12.8)	274 (13.3)	<0.000
Sex									
Male	263 (11.6)	238 (10.5)	297 (13.1)	316 (14)	256 (11.3)	232 (10.3)	374 (16.5)	287 (12.7)	<0.000
Female	788 (12.7)	788 (12.7)	716 (11.5)	760 (12.2)	799 (12.8)	905 (14.5)	738 (11.9)	729 (11.7)	<0.000
Marital status									
Never married	139 (19)	76 (10.4)	90 (12.3)	62 (8.5)	74 (10.1)	119 (16.3)	88 (12.1)	82 (11.2)	
Married	912 (11.8)	950 (12.3)	923 (11.9)	1014 (13.1)	981 (12.7)	1018 (13.1)	1024 (13.2)	934 (12)	<0.000
Highest level of educational attainment									
Tertiary	67 (18.3)	35 (9.6)	37 (10.1)	14 (3.8)	17 (4.6)	49 (13.4)	94 (25.7)	53 (14.5)	
Secondary	730 (14.3)	646 (12.6)	650 (12.7)	639 (12.5)	645 (12.6)	656 (12.8)	608 (12.8)	542 (10.6)	
Primary	248 (8.9)	328 (11.8)	302 (10.9)	391 (14.1)	376 (13.5)	363 (13.1)	389 (14)	382 (13.8)	
None	6 (2.7)	17 (7.6)	24 (10.7)	32 (14.2)	17 (7.6)	69 (30.7)	21 (9.3)	39 (17.3)	<0.000
Ethnic minorities									
MuslimMoor/Burgher	0	249 (15.3)	100 (6.2)	52 (3.2)	26 (1.6)	386 (23.7)	330 (20.3)	483 (29.7)	
Sinhalese	1(1)	9 (0.8)	189 (17.5)	0	0	0	680 (63)	201 (18.6)	
Tamil	1050 (18.2)	768 (13.3)	724 (12.6)	1024 (17.8)	1029 (17.9)	751 (13)	83 (1.4)	332 (5.8)	<0.000
Past displacement	265 (7.9)	434 (13)	311 (9.3)	999 (29.9)	927 (27.8)	129 (3.9)	7 (0.2)	268 (8)	<0.000
Hopkins Symptoms checklist									
Depression (> = 1.75)	411 (14.1)	403 (13.9)	338 (11.6)	501 (17.2)	460 (15.8)	381 (13.1)	114 (3.9)	302 (10.4)	<0.000
Anxiety (> = 1.75)	260 (13.9)	255 (13.7)	219 (11.7)	247 (13.2)	269 (14.4)	292 (14.4)	141 (7.6)	185 (9.9)	<0.000

^a^Column percentages are reported

### Prevalence of anxiety and depressive symptoms stratified by conflict-affected districts

Table 2 indicates that the prevalence of individual anxiety and depressive symptoms varied by district. The most widely endorsed symptoms across the conflict-affected districts included headaches (23%), feeling blue (22%), ongoing worries (20%), feeling everything is an effort (60%), and sense of worthlessness (53%) with populations in the severe conflict districts reporting higher prevalence of these symptoms compared to those in the moderate conflict districts.

**Table 2:** Prevalence (%) of anxiety and depressive symptoms across 8 conflict-affected districts (*n* = 8456) in Sri Lanka

		Jaffna *n* = 1051 (%)	Mannar *n =* 1026 (%)	Vavuniya *n =* 1013 (%)	Mullativu *n =* 1076 (%)	Killinochi *n =* 1055 (%)	Battcaloa *n =* 1137 (%)	Puttalam *n =* 1112 (%)	Trincomalee *n =* 1016 (%)	F-adj P	Total (*n =* 8456)
Anxiety symptoms											
1	Suddenly scared for no reason	36 (3.5)	57 (5)	47 (4.3)	28 (2.5)	31 (2.7)	68 (4.9)	22 (2.3)	50 (5.9)	0.0067	339 (3.6)
2	Feeling fearful	85 (8.3)	133 (11)	100 (9.4)	84 (7.8)	109 (9)	111 (9.4)	53 (4.7)	85 (10.1)	0.0013	760 (7.7)
3	Faintness, dizziness, or weakness	226 (22.8)	175 (17.4)	161 (15.2)	241 (22.4)	235 (21)	156 (12.8)	121 (9.4)	153 (17)	<0.0000	1468 (15.2)
4	Nervousness or shakiness inside	147 (14.1)	157 (14.4)	134 (12)	129 (12.4)	146 (12.9)	154 (12)	37 (3.6)	117 (11.3)	<0.0000	1021 (9.6)
5	Heart pounding or racing	148 (14.2)	141 (13.7)	122 (11.3)	155 (14.6)	150 (12.9)	140 (11.5)	52 (5)	113 (11.7)	<0.0000	1021 (10.1)
6	Trembling	136 (12.7)	153 (13.9)	135 (12.8)	109 (10.5)	150 (13.1)	137 (10.6)	37 (2.9)	117 (12.2)	<0.0000	974 (8.9)
7	Feeling tense or keyed up	110 (10.2)	128 (12.1)	111 (9.9)	106 (10.5)	109 (8.7)	131 (10.2)	43 (4.5)	104 (9.6)	<0.0000	842 (8.2)
8	Headaches	344 (30.8)	346 (32.3)	292 (25.7)	377 (33.1)	380 (33.6)	266 (20.7)	190 (12.8)	293 (25.8)	<0.0000	2488 (22.5)
9	Spells of terror or panic	10 (1.1)	11 (1.5)	24 (3.2)	22 (2.1)	19 (1.7)	32 (3.1)	35 (4.5)	32 (6.1)	0.0003	185 (3.3)
10	Feeling restless, can't sit still	102 (9.3)	72 (6)	67 (8.2)	109 (10.6)	129 (10.9)	66 (5.2)	61 (6.1)	77 (11.5)	0.015	683 (7.7)
Depressive symptoms											
11	Feeling low in energy—slowed down	171 (16.6)	183 (17.1)	150 (14.9)	174 (16.9)	166 (15.7)	115 (10.1)	97 (9)	127 (14.2)	0.0003	1183 (12.6)
12	Blaming yourself for things	120 (10.8)	113 (10.2)	82 (7.9)	129 (11.2)	153 (12.7)	114 (10)	34 (2.7)	95 (9.2)	<0.0000	840 (7.6)
13	Crying easily	194 (15.7)	232 (19.6)	154 (14)	226 (19.2)	258 (20.8)	195 (14.8)	27 (1.6)	153 (12)	<0.0000	1439 (10.7)
14	Loss of sexual interest or pleasure	30 (2.6)	34 (2.9)	50 (5.9)	21 (2.1)	30 (2.4)	45 (3.6)	35 (4.3)	63 (8.9)	0.012	308 (4.2)
15	Poor appetite	142 (13.3)	147 (13.4)	108 (10.1)	95 (8.3)	141 (12.3)	102 (8.2)	53 (4.1)	123 (10.7)	<0.0000	911 (8.7)
16	Difficulty falling asleep, staying asleep	211 (20.9)	218 (20.5)	180 (17.1)	204 (18.5)	219 (19.3)	206 (16.9)	80 (6.4)	157 (14.8)	<0.0000	1485 (14.2)
17	Feeling hopeless about the future	109 (9.7)	104 (8.8)	88 (7.8)	122 (11.2)	120 (10.7)	177 (16.8)	31 (2.6)	101 (10.1)	<0.0000	852 (8.2)
18	Feeling blue	359 (34.3)	358 (33.5)	284 (25.7)	485 (44.2)	460 (41.5)	236 (19.8)	129 (7.5)	255 (22)	<0.0000	2566 (21.5)
19	Feeling lonely	193 (17.6)	181 (16.5)	195 (18.3)	226 (20)	251 (21)	161 (13.5)	117 (7.3)	160 (14.9)	0.0001	1484 (13.3)
20	Feeling trapped or caught	25 (2.6)	16 (1.3)	15 (2)	27 (2.8)	29 (2.2)	32 (2.6)	14 (1.5)	20 (3.1)	0.126	178 (2.2)
21	Worrying too much about things	366 (33.6)	344 (31.7)	229 (20.9)	482 (43.7)	428 (38.6)	212 (17.2)	100 (6.5)	217 (19.5)	<0.0000	2378 (19.9)
22	Feeling no interest in things	67 (6.8)	86 (8.4)	74 (6.9)	65 (6)	65 (5.5)	97 (8.2)	29 (2.8)	72 (8.5)	0.0001	555 (5.8)
23	Thoughts of ending your life	30 (3)	42 (3.3)	42 (5)	19 (1.5)	19 (1.9)	55 (4.4)	18 (2)	40 (5.7)	0.0003	265 (3.2)
24	Feeling everything is an effort	953 (91.2)	915 (90)	746 (68.2)	1004 (94)	971 (92.2)	735 (64.2)	416 (26)	735 (61.8)	<0.0000	6475 (60)
25	Feelings of worthlessness	839 (81.5)	846 (82.3)	671 (62)	873 (82.9)	850 (82.4)	721 (64)	310 (16.2)	710 (59.2)	<0.0000	5820 (52.7)

^±^We defined “symptomatic depression” and “symptomatic anxiety” according to the conventional international cut-off scores of >1.75 for each subscale

### Joint invariance testing of 8 conflict-affected districts

Table 3 reports fit statistics for the configural models tested using different alignment optimization settings. The results showed that the bi-factorial model tested using the FIXED setting (with the moderate-minimal conflict zone as the reference category) indicated a good fit, supported by lowest values of AIC and BIC compared to the other models. Univariate residual analysis of the item pool showed that the adjusted residual values of all items fell within the 2 SDs from the mean, noting the small cell sizes due to the difference between the cell’s observed and expected frequency (with 13 items having a residual of less than −2) (Additional file 1).

**Table 3:** Fit statistics for invariance configural models tested using the alignment method across 8 conflict-affected districts

	Configural models	Alignment setting	No. of parameters	Log	AIC	BIC
1	Two-factor	Fixed	615	−18271.02	37772.04	42105.44
2	Three-factor	Fixed	623	−18901.53	39049.06	43438.93

*Log* loglikelihood, *AIC* Akaike, *BIC* Bayesian Information Criteria. Note: We tested a three-model based on the tripartite model proposed by Clark and Watson (1991) including the core constellations of anxiety-depressive symptoms and an additional domain of non-specific symptoms (insomnia, fatigue, restlessness, weakness, feeling tense). Models 1 and 2 tested using FREE method (all factor means were freely estimated) were poorly identified and therefore FIXED method (with Putalam fixed as the reference category) was used to estimate the model

The data suggest that, in spite of the expected level of non-invariance, it was possible to achieve measurement invariance of the HSCL-25 as a whole across eight conflict-affected districts.

The alignment analysis yielded an average metric (factor loading) non-invariance of 22.22% (well below the upper threshold of 25%). The districts that showed a relatively higher level of metric non-invariance (judged by the number of non-invariant metric parameters estimated in that group) included Mannar (number of invariant parameters = 3) and Putalam (*n =* 2), but again, these indices were well below the 25% threshold.

The analysis of scalar invariance yielded an average (intercept) scalar non-invariance of 5.88% (<25%). The districts that showed somewhat higher levels of scalar non-invariance (judged by the number of non-invariant scalar parameters estimated in that group) included Killinochi (*n =* 9), Mullativu (*n =* 8), Jaffna (*n =* 7), Mannar (*n =* 6) (Table 5).

Tables 4 and 5 report multigroup alignment analysis of metric and scalar invariance of the HSCL item pool across eight conflict affected districts. In comparison to the anxiety items, the depressive dimension showed a higher level of metric non-invariance, that is, the factor loadings associated with these items differed significantly across districts, included feeling blue, ongoing worries, feeling everything is an effort. Most anxiety and depressive items showed scalar invariance with the exception of the symptom of worthlessness. Table 6 reports the estimated alignment factor means based on the final model with metric and scalar invariance. The results indicated that the districts rank-ordered as having the highest anxiety mean scores are Jaffna, Mannar, Trincomalee, Killinochi, Mullativu, Batticola, Vavuniya, and Puttalam; and for depression, Trincomlaee, Jaffna, Batticola, Mannar, Mullativu, Killinochi, Vavuniya, and Puttalam. The additional configural models based on stratified samples of ethnic subgroups failed to converge.

**Table 4:** Metric invariance (factor loadings) for anxiety and depressive symptoms (numbers in parentheses refer to conflict-affected districts in Sri Lanka (*n* = 8456) that show significant non-invariance for the parameter)

		Jaffna (*n* = 1051)	Mannar (*n* = 1026)	Vavuniya (*n* = 1013)	Mullativu (*n* = 1076)	Killinochi (*n* = 1055)	Battcaloa (*n* = 1137)	Puttalam (*n* = 1112)	Trincomalee (*n* = 1016)
Anxiety symptoms									
1	Suddenly scared for no reason	1	2	3	4	5	6	7	8
2	Feeling fearful	1	2	3	4	5	6	7	8
3	Faintness, dizziness, or weakness	1	2	3	4	5	6	7	8
4	Nervousness or shakiness inside	1	2	3	4	5	6	7	8
5	Heart pounding or racing	1	2	3	4	5	6	7	8
6	Trembling	1	2	3	4	5	6	7	8
7	Feeling tense or keyed up	1	2	3	4	5	6	7	8
8	Headaches	1	2	3	4	5	6	7	8
9	Spells of terror or panic	(1)	(2)	3	4	5	(6)	7	8
10	Feeling restless, can't sit still	1	2	3	4	5	6	7	8
	Depressive symptoms	1	2	3	4	5	6	7	8
11	Feeling low in energy—slowed down	1	2	3	4	5	6	7	8
12	Blaming yourself for things	1	2	3	4	5	6	7	8
13	Crying easily	1	2	3	4	5	6	7	8
14	Loss of sexual interest or pleasure	1	2	3	4	5	6	7	8
15	Poor appetite	1	2	3	4	5	6	7	8
16	Difficulty falling asleep, staying asleep	1	2	3	4	5	6	7	8
17	Feeling hopeless about the future	(1)	(2)	(3)	4	5	6	7	8
18	Feeling blue	1	2	3	4	5	6	7	8
19	Feeling lonely	1	(2)	3	4	5	6	7	8
20	Feeling trapped or caught	1	2	3	4	5	6	7	8
21	Worrying too much about things	1	2	3	4	5	6	7	8
22	Feeling no interest in things	1	2	3	4	5	6	7	8
23	Thoughts of ending your life	1	2	3	4	5	6	7	8
24	Feeling everything is an effort	1	2	3	4	5	6	(7)	8
25	Feelings of worthlessness	1	2	3	4	5	6	(7)	8
	Total no. of non-invariant parameters	2	3	1	0	0	1	2	0

Degree of metric non-invariance = (25*8)/9 = 22.22

**Table 5:** Scalar invariance (intercepts) for aligned threshold parameters for anxiety and depressive symptoms (numbers in parentheses refer to conflict-affected districts in Sri Lanka (n = 8456) that show significant non-invariance for the parameter)

		Jaffna (n = 1051)	Mannar (n = 1026)	Vavuniya (n = 1013)	Mullativu (n = 1076)	Killinochi (n = 1055)	Battcaloa (n = 1137)	Puttalam (n = 1112)	Trincomalee (n = 1016)
Anxiety symptoms									
1	Suddenly scared for no reason	1	2	3	4	5	6	7	8
2	Feeling fearful	1	2	3	4	5	6	7	8
3	Faintness, dizziness, or weakness	(1)	2	3	4	(5)	6	7	8
4	Nervousness or shakiness inside	1	2	3	4	5	6	7	8
5	Heart pounding or racing	1	2	3	4	5	6	7	8
6	Trembling	1	2	3	4	5	6	7	8
7	Feeling tense or keyed up	(1)	2	3	4	5	6	7	8
8	Headaches	1	(2)	3	(4)	(5)	6	7	8
9	Spells of terror or panic	(1)	2	3	(4)	(5)	6	7	8
10	Feeling restless, can't sit still	1	(2)	3	4	5	(6)	7	8
	Depressive symptoms	1	2	3	4	5	6	7	8
11	Feeling low in energy--slowed down	1	2	3	4	(5)	6	7	8
12	Blaming yourself for things	1	2	3	4	5	6	7	8
13	Crying easily	1	2	3	4	5	6	(7)	8
14	Loss of sexual interest or pleasure	1	(2)	3	(4)	5	6	7	8
15	Poor appetite	1	2	3	4	5	6	7	8
16	Difficulty falling asleep, staying asleep	1	2	3	4	5	6	7	8
17	Feeling hopeless about the future	1	2	3	4	5	6	(7)	8
18	Feeling blue	(1)	(2)	3	(4)	(5)	6	7	8
19	Feeling lonely	1	2	3	4	(5)	6	7	8
20	Feeling trapped or caught	1	2	3	4	5	6	7	8
21	Worrying too much about things	(1)	2	3	(4)	(5)	6	7	8
22	Feeling no interest in things	1	2	3	4	5	6	7	8
23	Thoughts of ending your life	1	2	3	4	5	6	7	8
24	Feeling everything is an effort	(1)	(2)	3	(4)	(5)	6	(7)	8
25	Feelings of worthlessness	(1)	(2)	3	(4)	(5)	6	(7)	8
	Total no. of non-invariant parameters	7	6	0	7	9	1	4	0

Degree of scalar non-invariance = (25*8)/34 = 5.88

**Table 6:** Comparisons of factor means of anxiety and depressive symptoms across 8 conflict-affected districts in Sri Lanka (*n* = 8456) (factor means are rank-ordered and significant at *P* = 0.05)

Ranking	Group	Factor mean
Anxiety symptoms		
1	1 (Jaffna)	0.704
2	2 (Mannar)	0.692
3	8 (Trincomalee)	0.642
4	5 (Killinochi)	0.638
5	4 (Mullativu)	0.626
6	6 (Batticola)	0.550
7	3 (Vavuniya)	0.542
8	7 (Puttalam)	0.100
Depressive symptoms		
1	8 (Trincomalee)	0.721
2	1 (Jaffna)	0.697
3	6 (Batticola)	0.691
4	2 (Mannar)	0.690
5	4 (Mullativu)	0.655
6	5 (Killinochi)	0.599
7	3 (Vavuniya)	0.571
8	7 (Puttalam)	0.100

^a^Factor means representing each subscale were generated and compared on the basis of scalar (intercept) invariance.

## Discussion

Our study tested the bifactorial structure and the measurement invariance of the HSCL-25 across eight conflict-affected districts, with the moderate-minimal conflict area (Putalam) fixed as the reference group. The bifactorial model outperformed the tripartite model. Our findings therefore provide support for the bifactorial model in which the HSCL items were divided into the clinically conventional dimensions of anxiety and depressive symptoms, a common structure identified across past studies in the post-conflict field [13, 14, 17, 26]. Our findings provide the first analysis of the measurement invariance of the HSCL in subpopulations across a broad range of geographic regions using a novel statistical method. Our findings show that, in spite of the level of non-invariance identified in the HSCL items, an expected outcome in transcultural measurement testing [3], it was possible to achieve invariance for the anxiety and depression dimensions of the measure across a number of conflict-affected groups that differ in geographical location. A further validation of our findings was that populations residing in the most severe conflict areas reported a substantially higher prevalence of anxiety and depression compared to moderate and minimal conflict areas, a pattern that is broadly consistent with our recent analysis of the same dataset, thereby attesting to the construct validity of anxiety-depression at least in this population [45]. The severe conflict districts (Mannar, Killinochi, Mullativu) were heavily populated by ethnic minorities including Tamils, Moors, and Burghers. Configural models based on stratified samples of ethnic subgroups failed to converge, a finding that might be attributable to the low representation of ethnic subgroups across some conflict-affected districts.

In comparison to the anxiety items, the depressive dimension showed relatively higher levels of metric non-invariance with the factor loadings associated with several items (feeling blue, ongoing worries, feeling everything is an effort) differing significantly across districts. By far the majority of anxiety and depressive items showed scalar invariance, the exception being the symptom of worthlessness.

Our study is the first to employ the multigroup alignment method in this field, a novel statistical approach for conducting joint invariance testing across a substantial number of groups, in this instance eight districts stratified by regional conflict exposure and which differ in ethnic composition. In addition, the sample size was large and was from the largest study in the post-conflict field in general and Sri Lanka in particular. The alignment method offers greater flexibility compared to conventional Multigroup CFA in that the former relaxes the restrictive nature of iterative testing of metric and scalar invariance by applying the configural model in a manner that automatically identifies the optimal solution based on the minimal degree of non-invariance in all relevant measurement parameters [7].

Our analysis identified several items of the depression and anxiety scales as showing the greater degree of scalar non-invariance including feeling blue, ongoing worries, feeling everything is an effort, and worthlessness. These findings are consistent with other studies that found variations in depression scores yielded by different instruments across culturally diverse communities such as the Korean [46], Japanese [47], Chinese [48] populations, with lower intercepts generally being recorded amongst the East Asian communities who exhibit a tendency towards emotional or affective suppression.

Our findings indicate that there may be substantial variations in the manner that some symptoms of depression and anxiety are understood and interpreted across geographically dispersed populations with different ethnic and language distributions, suggesting that these items may not be as robust in representing the emotional status of the Sri Lankan society as a whole [49, 50]. Specifically, past studies found greater prevalence of somatic symptoms relative to psychological or behavioural symptoms amongst individuals presented with depressive and anxiety disorders, suggesting that the reaction patterns differ to some extent across cultures [51–54]. It is plausible therefore that the items identified in our analysis as being non-invariant may correspond more closely to the western construct of depression.

The alignment method used in our analysis is a novel approach that allows for joint invariance testing of a large number of groups. Our study is the first in the post-conflict field to test the bifactorial structure and measurement invariance of the anxiety and depression dimensions of the HSCL-25. In undertaking the analysis, we drew on a nation-wide survey of Sri Lankan populations stratified across regions exposed to severe and moderate levels of conflict. Nevertheless, there are limitations in this study. Although previously used in research in Sri Lanka, it is acknowledged that the HSCL-25 was not re-calibrated against a gold standard clinical interview amongst all ethnic subpopulations studied.

Establishing measurement invariance across ethnic and linguistic groups living in different geographic regions is important for both theoretical and practical reasons. There is a long legacy of debate focusing on the transcultural equivalence of mental health categories such as depression and anxiety across culturally distinct communities [52, 55–58]. Critics of the notion of universality argue that diagnoses such as these are culture bound and do not necessarily correspond with concepts of suffering across diverse cultures [59, 60]. From a pragmatic perspective, even if an assumption of universality is adhered to, consideration needs to be given to the influence of culture, history, language, and religion in shaping understandings of mental disorder categories and the way individuals from different groups may respond to systematic inquiries into the symptoms that constitute a particular category. In relation to the HSCL, past studies have shown that measures of depression [27, 61] and anxiety (including PTSD prevalence measured using the HTQ, the most widely used measure in the field [3]) have yielded considerable variations in scores across diverse populations, raising questions about the construct equivalence of these categories across diverse cultures; it is therefore imperative that cross-cultural measures such as the HSCL are adapted to the culture, context, language, and characteristics of each community. In adapting psychiatric measurement tools, researchers in the field have applied mixed-method approaches grounded in etic and emic perspectives [62–65], drawing on qualitative data gathered from key informant interviews and focus groups, including locally salient terms and expressions that correspond to categories specified in the contemporary diagnostic systems.

Caution needs to be exercised in concluding that ethnicity did not influence the pattern of invariance of the measure, particularly given that the small numbers of some ethnic minorities included (Burghers, Moors) meant that they had to be conflated into a single composite group, a possible reason why there was non-convergence of the model testing invariance by ethnicity. As such, our findings should be interpreted only as indicating broad validation of the construct and ecological validity of the HSCL-25 across conflict-affected populations in Sri Lanka. Finally, while the use of the alignment method is novel in the field of transcultural mental health and it allows for joint-testing of invariance across a large number of groups, further testing and simulation studies based on other samples are required to establish accurate fit indicators and modification indices that can be applied to invariance model testing and refinement. The alignment analysis requires a configural model to be specified correctly prior to further alignment of parameters, minimizing the otherwise cumbersome procedure of iterative model modification and respecification in conventional MG-CFA, particularly when testing the invariance of a large number of items across multiple groups.

## Conclusions

A novel aspect of our study is that it is the first in the post-conflict field and in psychiatry in general to employ the alignment method to examine the invariance of the HSCL in a nation-wide epidemiological survey undertaken in a country seven years following an extensive period of conflict that affected large sectors of the Sri Lankan population. Our findings provided a foundation on which future studies may apply the alignment method especially when testing measurement variance and construct validity of psychiatric measures across a large number of culturally diverse groups.

Our findings demonstrate the methodological feasibility of applying the alignment method to test the structure and invariance of the HSCL across ethnically diverse populations living in conflict-affected districts in Sri Lanka. In addition, our findings provide additional support for the HSCL as a screening measure for broadly defined symptoms of anxiety and depression both in community and clinical settings in non-western populations. For example, the HSCL may be applied as a general measure for monitoring population trends, particularly in relation to ecological and social correlates of anxiety and depression, and how changes in the former may influence the trajectory of the latter over time. The data gathered may be valuable in informing public policy in relation to identifying the types of programs that may assist in reducing anxiety and depression in the community as a whole. Given that the failure of convergence of our alignment model may be related to the low representation of ethnic minorities within our survey, further studies are needed to examine ethnicity and language factors more critically. Finally, future calibration studies are needed to examine the concurrent validity of the HSCL, based on comparisons with a gold standard clinical interview conducted across all ethnic groups, to ensure that context-specific case thresholds for the HSCL are applied in clinic settings.

## Additional file

Additional file 1: Table S7. Adjusted residuals of items of depression and anxiety scales of the HSCL-25. (DOCX 13 kb)
