# Supplementary material for: Measurement invariance of the Hopkins Symptoms Checklist: a novel multigroup alignment analytic approach to a large epidemiological sample across eight conflict-affected districts from a nation-wide survey in Sri Lanka
Source: Confl Health. 2017 Apr 26;11:8. doi: 10.1186/s13031-017-0109-x (PMC5405490; doi:10.1186/s13031-017-0109-x)
Supplement: Additional file 1: Table S7. — Adjusted residuals of items of depression and anxiety scales of the HSCL-25. (DOCX 13 kb) [file 13031_2017_109_MOESM1_ESM.docx]

Table 7 (supplementary file). Adjusted residuals of items of depression and anxiety scales of the HSCL-25.

|  | **Anxiety symptoms** | Observed | Estimate | Adjusted residuals |
| --- | --- | --- | --- | --- |
| 1 | Suddenly scared for no reason | 0.035 | 0.035 | 0.000 |
| 2 | Feeling fearful | 0.083 | 0.082 | 0.001 |
| 3 | Faintness, dizziness, or weakness | 0.228 | 0.224 | 0.005 |
| 4 | Nervousness or shakiness inside | 0.141 | 0.14 | 0.001 |
| 5 | Heart pounding or racing | 0.142 | 0.14 | 0.001 |
| 6 | Trembling | 0.127 | 0.127 | 0.000 |
| 7 | Feeling tense or keyed up | 0.102 | 0.104 | -0.002 |
| 8 | Headaches | 0.308 | 0.301 | 0.006 |
| 9 | Spells of terror or panic | 0.011 | 0.011 | 0.000 |
| 10 | Feeling restless, can't sit still | 0.93 | 0.092 | 0.001 |
|  | **Depressive symptoms** |  |  |  |
| 11 | Feeling low in energy--slowed down | 0.165 | 0.227 | -0.061 |
| 12 | Blaming yourself for things | 0.108 | 0.152 | -0.044 |
| 13 | Crying easily | 0.157 | 0.213 | -0.056 |
| 14 | Loss of sexual interest or pleasure | 0.026 | 0.038 | -0.012 |
| 15 | Poor appetite | 0.133 | 0.193 | -0.06 |
| 16 | Difficulty falling asleep, staying asleep | 0.209 | 0.284 | -0.074 |
| 17 | Feeling hopeless about the future | 0.097 | 0.093 | 0.003 |
| 18 | Feeling blue | 0.343 | 0.429 | -0.086 |
| 19 | Feeling lonely | 0.176 | 0.237 | -0.06 |
| 20 | Feeling trapped or caught | 0.026 | 0.031 | -0.005 |
| 21 | Worrying too much about things | 0.336 | 0.415 | -0.079 |
| 22 | Feeling no interest in things | 0.068 | 0.103 | -0.035 |
| 23 | Thoughts of ending your life | 0.03 | 0.042 | -0.012 |
| 24 | Feeling everything is an effort | 0.912 | 0.91 | 0.003 |
| 25 | Feelings of worthlessness | 0.814 | 0.786 | 0.029 |
